# Maf1-mediated regulation of yeast RNA polymerase III is correlated with CCA addition at the 3′ end of tRNA precursors

**DOI:** 10.1016/j.gene.2016.08.033

**Published:** 2017-05-15

**Authors:** Dominika Foretek, Przemysław Nuc, Marek Żywicki, Wojciech M. Karlowski, Grzegorz Kudla, Magdalena Boguta

**Affiliations:** a Department of Genetics, Institute of Biochemistry and Biophysics, Polish Academy of Sciences, 02-106 Warsaw, Poland; b Institute of Molecular Biology and Biotechnology, Adam Mickiewicz University, 61-614 Poznan, Poland; c Department of Computational Biology, Institute of Molecular Biology and Biotechnology, Faculty of Biology, Adam Mickiewicz University, 61-614 Poznan, Poland; d MRC Human Genetics Unit, IGMM, University of Edinburgh, Edinburgh, Scotland, UK

**Keywords:** Pol III, RNA polymerase III, tRNA, transfer RNA, pre-tRNA, precursor tRNA, *maf1*Δ, strain with deleted gene encoding Maf1, *Saccharomyces cerevisiae*, Pre-tRNA, tRNA processing, tRNA transcription, RNA-seq, tRNA nucleotidyltransferase

## Abstract

In eukaryotic cells tRNA synthesis is negatively regulated by the protein Maf1, conserved from yeast to humans. Maf1 from yeast *Saccharomyces cerevisiae* mediates repression of trna transcription when cells are transferred from medium with glucose to medium with glycerol, a non-fermentable carbon source. The strain with deleted gene encoding Maf1 (*maf1*Δ) is viable but accumulates tRNA precursors. In this study tRNA precursors were analysed by RNA-Seq and Northern hybridization in wild type strain and *maf1*Δ mutant grown in glucose medium or upon shift to repressive conditions. A negative effect of *maf1*Δ mutant on the addition of the auxiliary CCA nucleotides to the 3′ end of pre-tRNAs was observed in cells shifted to unfavourable growth conditions. This effect was reduced by overexpression of the yeast *CCA1* gene encoding ATP(CTP):tRNA nucleotidyltransferase. The CCA sequence at the 3′ end is important for export of tRNA precursors from the nucleus and essential for tRNA charging with amino acids. Data presented here indicate that CCA-addition to intron-containing end-processed tRNA precursors is a limiting step in tRNA maturation when there is no Maf1 mediated RNA polymerase III (Pol III) repression. The correlation between CCA synthesis and Pol III regulation by Maf1 could be important in coordination of tRNA transcription, processing and regulation of translation.

## Highlights

• Effect of Maf1 on maturation of tRNA precursors was analysed in yeast cells.
• CCA addition to the 3′ end of pre-tRNA was down-regulated in *maf1*Δ mutant under stress.
• Effect of inactivation and overproduction of Cca1 enzyme in *maf1*Δ cells was examined.
• Link between CCA synthesis and RNA polymerase III regulation is discussed.

## 1 Introduction

The nuclear genome of *Saccharomyces cerevisiae* contains 274 tRNA genes dispersed across all chromosomes, all of which are actively transcribed by RNA polymerase III (Pol III) (Chan and Lowe, 2009, Hani and Feldmann, 1998). The reported lengths of the primary pre-tRNA transcripts vary between 72 and 145 nt (O'Connor and Peebles, 1991), where around 20% of tRNA genes contain introns. Additionally tRNAs are grouped into 20 isotypes, each charged with a single type of amino acid, which are subdivided into 41 isoacceptors that each recognize the same anticodon sequence(s) (Hani and Feldmann, 1998).

Maturation of initial transcripts generates a variety of stable and unstable tRNA species.

Generally the first step in tRNA maturation is 5′ leader cleavage by RNase P (Xiao et al., 2001), followed by 3′ end processing by Trz1 endonuclease and Rex1, a 3′-5′ exonuclease (Ozanick et al., 2009, van Hoof et al., 2000). tRNA 3′ end-processing also involves Lhp1, a homologue of human La protein which stabilizes pre-tRNAs by direct binding to the oligo U tract at the 3′ termini, generated upon Pol III transcription termination (Lin-Marq and Clarkson, 1995, Stefano, 1984). By interaction with Lhp1 a complex of Lsm proteins may also play a role in tRNA end processing (Kufel et al., 2002). Finally, under some conditions Rrp6, a 3′-5′ exonuclease that is a component of the nuclear exosome, performs tRNA 3′ end processing which appears to compete with pre-tRNA degradation (Maraia and Lamichhane, 2011, Gudipati et al., 2012, Skowronek et al., 2014). Following removal of the 3′ trailer from the original transcript, the ubiquitous CCA nucleotides are added to the 3′ terminus by tRNA-nucleotidyltransferase Cca1 (Aebi et al., 1990). After end-maturation followed by nuclear export, splicing of intron-containing precursors occurs in the cytoplasm. Throughout all the maturation steps modifications are added; the modified nucleotides serve numerous functions including an important role in maintaining proper tRNA structure and stability (Phizicky and Hopper, 2010).

Pol III transcription rate is tightly coupled to environmental conditions and is regulated by the highly conserved repressor Maf1 (Boguta, 2013). A yeast strain with deleted gene encoding Maf1 (*maf1*Δ) is viable but accumulates tRNA precursors (Karkusiewicz et al., 2011). Several different signalling pathways have been reported to induce Maf1 dependent repression. Notably, Maf1 represses Pol III when cells are transferred from glucose containing medium to media with non-fermentable carbon sources, such as glycerol, and elevated temperature (37 °C) (Ciesla et al., 2007). Additionally, we have shown that respiratory growth and elevated temperature down-regulate 3′-end processing of tRNA precursors and at least for two tRNAs different steps of their maturation are affected (Foretek et al., 2016).

To better characterize the overall effect of *maf1*Δ, temperature and carbon source on tRNA processing we employed the Illumina platform for RNA-Seq analysis, to precisely assess the 3′-ends of pre-tRNAs. Libraries for high-throughput sequencing of tRNA-precursors were prepared from RNA isolated from wild type and *maf1*Δ mutant cells grown under permissive and restrictive conditions. Sequencing results were compared to patterns of tRNA precursors detected by Northern analyses. The most interesting observation which we further explored was the effect of Maf1 and growth conditions on CCA-addition to 3′ termini of pre-tRNA.

## 2 Materials and methods

### 2.1 Strains, plasmids, and media

Yeast strains BY4741 (*MAT*α *his3*Δ*1 leu2*Δ*0 met15*Δ*0 ura3*Δ*0*) and *rex1*Δ were obtained from the yeast deletion collection (Open Biosystems). *maf1*Δ (BY4742: *MAT*α *his3*Δ*1 leu2*Δ*0 lys2*Δ*0 ura3*Δ*0 maf1*::*KanMX4*) was obtained from Euroscarf. *cca1-1* (in BY4741 background) was obtained from A. K. Hopper laboratory and *cca1-1 maf1*Δ was prepared in this study by introducing the *maf1*Δ ∷* URA3* cassette to *cca1-1* strain. The plasmid harboring *CCA1* derived from the Yeast Genomic Tiling Collection (GE)(KanMx, LEU2, 2 μ) (Jones et al., 2008). pRS425 plasmid was used as the control vector (Christianson et al., 1992).

YPD or YPGly complete media contained 1% yeast extract (Fisher Scientific), 1% peptone (BD), and 2% glucose (Fisher Scientific) or 2% glycerol (Sigma), respectively. Minimal medium (SC) contained 2% glucose and 0.67% yeast nitrogen base (BD). SC-leu medium contained supplements required for growth (at 20 μg/ml each, except leucine).

### 2.2 Northern blot analysis

Overnight yeast cultures were re-suspended in fresh YPD or SC-leu media to OD_600_ = 0.15 and grown to exponential phase (OD_600_ = 0.5–0.8) at 30 °C or 23 °C (for thermo-sensitive strains). Cells were collected by centrifugation, washed and re-suspended in YPGly media and incubated at 37 °C. After incubation under stress conditions for 2 h, the cells were harvested, washed and re-suspended in TSE buffer (0.01 M Tris, 0.01 M EDTA, 0.1 M NaCl, pH 7.5). RNA isolation and Northern Hybridization was done as described previously (Foretek et al., 2016).

For sequencing resolution gel the 6% polyacrylamide (acrylamide: bisacrylamide 19:1) and 8 M urea were used. 10 μg of RNA was loaded on the gel and run in 0.5 × TBE buffer (44.5 mM Tris (pH 7.6), 44.5 mM boric acid, 1 mM EDTA) for 3–4 h at 35 W, followed by transfer onto positively charged nylon membranes for 1.5 h at 200 mA in 0.5 × TBE. All subsequent steps were done as described previously (Foretek et al., 2016).

For RNA hybridization the following DIG-labeled oligonucleotides were used: JW0011 tI(UAU) 5′-GGCACAGAAACTTCGGAAACCGAATGTTGCTATAAGCACGAAGCTCTAACCACTGAGCTACA-3′;tK(UUU) 5′-ATCCTTGCTTAAGCAAATGCGCT-3′, tL(CAA) 5′-TATTCCCACAGTTAACTGCGGTCA-3′, 5.8S rRNA 5′-GCGTTGTTCATCGATGC-3′.

### 2.3 Library preparation and Illumina sequencing

Yeast cultures and RNA isolation were prepared as for the Northern hybridization. 700 ng of each RNA was ligated with 42 pmole of 3′-tRNA adapter using T4 RNA Ligase (Ambion) for 2 h at room temperature. After ligation RNA was phenol: chloroform extracted and ethanol precipitated with addition of 1 μl of Glycoblue (Ambion). Total RNA with adapter was re-suspended in water and reverse transcribed with 60 pmol of RT/PCR primer 1 (specific to adapter sequence) and Super Script II Reverse Transcriptase (Life Technologies) according to manufacturer's protocol with incubation time of 1.5 h at 42 °C. The cDNA was amplified in PCR reaction using Phusion Hot Start Flex DNA Polymerase (NEB), 25 pmol of PCR primer 1 and mixture of tF(GAA)N, tK(UUU)D, tL(CAA)A, tW(CCA)G1 primers (3.125 pmol each) and subsequent program: initial denaturation 98 °C for 2 min, followed by 17 cycles of 30 s at 98 °C, 30 s at 55 °C, and 30 s at 72 °C and final extension in 72 °C for 5 min. PCR products were phenol: chloroform extracted and ethanol precipitated with addition of glycoblue. Resuspended DNA was loaded on 10% polyacrylamide (acrylamide: bisacrylamide 29:1) 8 M urea gel with 10 bp ladder (Life Technologies) and run in 1 × TBE buffer (89 mM Tris (pH 7.6) 89 mM boric acid 2 mM EDTA) under ~ 300 V for 2–3 h. The gel was stained with Sybr Gold (Life Technologies) and bands corresponding to size between 65 and 135 bp were cut out from the gel followed by DNA elution with elution buffer (50 mM Mg-acetate, 0.5 M ammonium acetate, 1 mM EDTA, 0.1% SDS) with shaking at room temperature overnight. Next, DNA was ethanol precipitated with glycoblue and RNAse A (Sigma) treated according to manufacturer's protocol for removal of residual RNAs. One μg of PCR product was used for second PCR using: 2-nd PCR primer and mixture of tF(GAA)N ext., tI(UAU)L ext., tK(UUU)D ext., tL(CAA)A ext., tW(CCA)G1 and the same PCR cycling conditions as in the first reaction, reducing the amplification step to 10 cycles. Phenol: chloroform extracted DNA was run on 10% polyacrylamide (acrylamide: bisacrylamide 29:1) 8 M urea gel and the fragments corresponding to size between 100 and 180 bp were eluted from the gel. 3 ng of the DNA template obtained was used for a final PCR reaction with primers containing barcodes for Illumina sequencing platform – “Universal 5′ final PCR” and RP1 ÷ RP6 for each sample respectively. The PCR cycling program consisted of following steps: 98 °C for 2 min, 11 cycles of 30 s at 98 °C, 30 s at 60 °C, and 30 s at 72 °C and final extension in 72 °C for 7 min. The reaction product was phenol: chloroform extracted and ethanol precipitated with glycoblue and run on a native gel (10%, 29:1 acrylamide: bisacrylamide with 1% glycerol) and run at 125 V for 2 h. After Sybr Gold staining the product in a size range of 156–236 bp was cut out, quality controlled on Qubit and sent to FASTERIS SA where high-throughput sequencing was performed. As additional control around 100 ng of DNA from each sample was ligated into pGEM vector (as in 3′ end ligation and sequencing procedure) and 10 clones for each library were sequenced to check if they contained proper PCR constructs.

### 2.4 High-throughput sequencing data analysis

Six libraries were run on a HiSeq 2000 Illumina platform in HiSeq Flow Cell v3 and TruSeq SBS Kit v3. First quality filtering was performed by the FASTERIS SA company using HiSeq Control Software 2.0.5, RTA 1.17.20.0 and CASAVA-1.8.2 software. Only reads with quality score equal or higher than 30 were further analysed. First, flexbar (Dodt et al., 2012) was applied to remove the 5′ linker with options – f sanger (format) – as TGGAATTCTCGGGTGCCAAGGC (adapter sequence) – ao 4 (minimal number of bp to be removed) – m 15 (minimum read length to remain after removal) – n 16 (number of threads to employ). Next the reads were converted to fasta format, reverse complemented and the identical reads were collapsed using custom scripts. The data obtained were aligned using blastall program version 2.2.25 with parameters – p blastn (nucleotide to nucleotide comparison) – e 0.001 (cutoff value) – W 8 (word size) – m 8 (output format) – G 2 (cost to open gap) – E 1 (cost of extending gap) – F F (filtering) and yeast genome database was downloaded from USCS (https://genome.ucsc.edu/), version sacCer3 (S288C reference genome from 2011). The data obtained from BLAST alignment and custom prepared scripts were used to analyse all sequences and genomically non-encoded 3’ends (like oligo A tails and CCA) using tools described in (Tuck and Tollervey, 2013). The number of reads for each sequence or gene was normalized and presented as a percentage of all reads aligned to particular tRNA in a library.

## 3 Results

### 3.1 Processing of pre-tRNAs is altered in *maf1*Δ cells

**Fig. 1:**
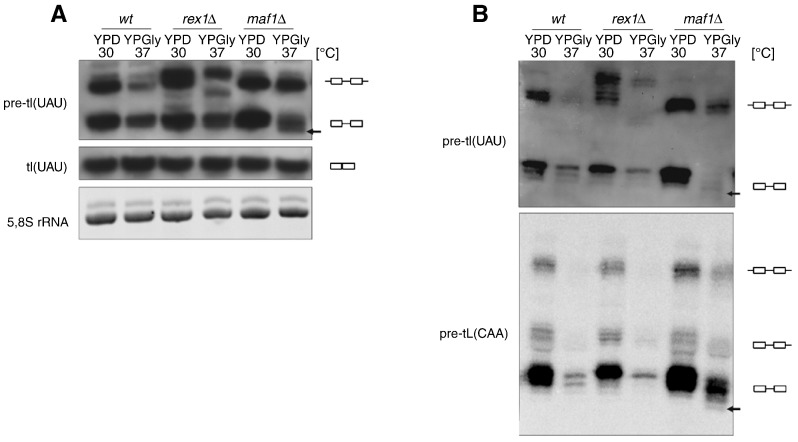
tRNA processing is altered in *maf1*Δ mutant. Yeast were grown as described in text and harvested. Isolated RNA was separated on polyacrylamide gel (A) or sequencing gel (B) followed by Northern analysis with specific probes.

A previous study identified that both primary transcripts and end-processed intron-containing tRNA precursors accumulate in *maf1*Δ mutant (Karkusiewicz et al., 2011). To further evaluate the effect of *maf1*Δ on processing of tRNA precursors we employed Northern hybridization. Yeast grown to exponential phase in complete glucose medium (YPD) were shifted for 2 h to media with glycerol as the carbon source (YPGly) and elevated temperature (37 °C). An isogenic *rex1*Δ strain lacking Rex1 exonuclease involved in processing of 3′ end of initial tRNA transcript was used as an additional control, because it has been shown before to have a strong phenotype in the conditions employed (Foretek et al., 2016). The processing of intron-containing tI(UAU), was monitored by hybridization with a specific probe (Fig. 1A). As expected, the level of both: unprocessed initial transcripts (designated ) as well as end-matured intron-containing pre-tRNAs (designated ) were increased in *maf1 ∆* mutants relative to wild type cells. The aberrant intermediates, which accumulate in *rex1*Δ were observed as well, in agreement with previous report (Foretek et al., 2016). Noteworthy, a fraction of the end-processed intron-containing precursors of tI(UAU), migrate faster in *maf1*Δ than in other tested strains (Fig. 1A, black arrow). For precise detection of all intermediates of tRNA maturation, RNA was resolved on sequencing resolution gel prior to Northern analysis (Fig. 1B). Clearly, the faster migrating forms of two representative intron-containing tRNAs, tL(CAA) and tI(UAU), were detectable in *maf1*Δ cells shifted to restrictive conditions (YPGly 37 °C). We thus concluded that Maf1 affects tRNA processing in specific manner.

### 3.2 High throughput sequencing of tRNA precursors

To confirm and extend the analysis of the effect of *maf1*Δ on maturation of other pre-tRNAs, high-throughput sequencing of tRNA precursors was performed. We took under consideration the observation that alterations of growth temperature and carbon source specifically affected 3′ end processing of primary transcripts (Foretek et al., 2016). Additionally, the size of the lower migrating form in *maf1*Δ strain correlated with the size of 5′ end processed intron-containing pre-tRNAs. Together this led to the hypothesis that undefined forms of pre-tRNAs detected in *maf1*Δ strain (Fig. 1) differ at their 3′ terminus and we further studied it on a larger scale.

Five pre-tRNAs were selected for the RNA-Seq: tI(UAU), tF(GAA), tL(CAA), tW(CCA) and tK(UUU), based on our recent analysis (Foretek et al., 2016) and previous Pol III microarray data which showed highly upregulated transcription of four of selected tRNAs in *maf1*Δ under stress (Ciesla et al., 2007).

RNA used for high-throughput sequencing on the HiSeq 2000 Illumina platform was isolated from wild type, *maf1*Δ and *rex1*Δ strains grown in standard conditions to exponential phase and transferred for 2 h to YPGly media at 37 °C. Since tRNA precursors are much less abundant than mature tRNAs, double selection has been applied to amplify halves of pre-tRNAs, starting from the intron and ending at the 3′ termini. First a 3′ adapter was ligated to the 3′ ends of total RNAs and reverse transcribed with primer specific to the adapter. Next, a mixture of primers with sequences complementary to the introns of five selected pre-tRNA was used to only amplify the designated pre-tRNAs followed by gel purification of products of selected size and barcoding to generate cDNA library for high-throughput sequencing. Separate libraries were prepared for wild type, *maf1*Δ and *rex1*Δ cells grown in standard and stress conditions (YPD 30 °C and YPGly 37 °C respectively) (see M&M). For each library 1.4 to 5.2 million single-end 100 nucleotide reads were obtained. Using customized scripts all reads were tested if their sequences were fully aligned to the genomic sequence for a particular tRNA gene or if they possessed additional nucleotides at their 3′ termini. At least 96% of the processed reads were aligned to yeast tRNA genes. Total number of reads mapped to each pre-tRNA in each library was used in calculation of percentage of each designated group of reads. The proportions of reads corresponding to trimmed tRNAs or intron-containing tRNA precursors, processed or unprocessed at 3’ends, are listed in Table S1. These data show that efficiency of 3′ end processing is dependent on growth conditions but mostly unaffected by Maf1 (except tK(UUU)). All reads aligned to pre-tRNAs and harboring posttranscriptional modification at 3′ end by oligoadenylation or CCA-addition were classified in Table 1. Full or partial CCA triplet was taken into consideration only when it was following the end of tRNA gene.

The known substrates of polyadenylation are unprocessed tRNA precursors or tRNA fragments designated for degradation (Wlotzka et al., 2011, Schneider et al., 2012, Gudipati et al., 2012) and both types have been identified by RNA-Seq analysis (Table 1). In the wild type strain under standard conditions we detected 2–14% (depending on tRNA) of reads with one or more extra A at 3′ end which indicate their turnover by exosomes (Jia et al., 2011, Jia et al., 2012). In *rex1*Δ mutant, tI(UAU), tL(CAA) and tW(CCA) have increased oligoadenylation, in agreement with previously published data (Ozanick et al., 2009). Interestingly, for majority of pre-tRNAs oligoadenylation is slightly increased under stress conditions.

The efficiency of post-transcriptional CCA modification at 3′ end varied for different tRNAs (Table 1). Under standard conditions in wild type strain the most efficient modification was observed for tK(UUU) with 44% of reads having full CCA sequence added at their 3′ end. In contrast, tF(GAA) tRNA had only 2.5% of reads with CCA and over 85% of reads aligned to the genomic sequence including trailer (Table 1 and Table S1). Surprisingly, the efficiency of CCA addition was differently affected by stress for individual tRNAs – substantially reduced for tK(UUU) (from 44% to 3.3%) but increased for tL(CCA) (from 29.4% to 51.4%). Interestingly, the relative amount of intermediate forms with C- and CC at 3′ end also varied among the tRNAs examined. Perhaps it reflects the rate of CCA synthesis, which might be dependent on the conformation of 3′ end. The *rex1*Δ mutant strain does not show any defined trend of influence on CCA addition for all tested pre-tRNAs when compared to the wild type from both growth conditions. However, the *maf1*Δ cells have a lower percentage of precursors with full CCA extensions for tI(UAU), tL(CAA) and tW(CCA) in comparison to wild type cells shifted to stress conditions. Additionally, in *maf1*Δ, tI(UAU), tK(UUU), tL(CAA) and tW(CCA) under restrictive conditions have more oligoadenylated pre-tRNAs. This indicates that overproduction of pre-tRNAs in *maf1*Δ negatively affects tRNA processing and CCA addition but it should be noted that, different pre-tRNAs are affected to a different extent.

**Fig. 2:**
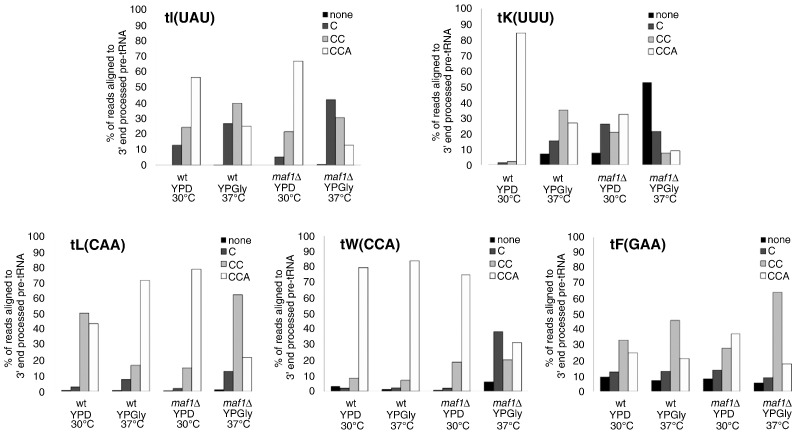
Efficiency of CCA addition to 3′ end of tRNA precursors is decreased in cells shifted to stress conditions. The bars represent RNA-seq data for given libraries from wild type and *maf1*Δ strains. The number of reads for each of presented pre-tRNA forms was normalized to all the reads mapped to the 3′ end processed tRNA and presented as a percentage of the reads for given pre-tRNA.

To explore the effect of *maf1*Δ on CCA addition, the class of reads corresponding only to intron-containing 3′ end-matured tRNA precursors was separately analysed (Fig. 2). The relative ratios of reads corresponding to precursors with extra C, CC or CCA added at the 3′ ends were increased as compared to data presented in Table 1 where tRNA fragments and pre-tRNAs containing trailers were included in the total number of reads for percentage calculation. This ratio where only end-processed precursors are taken into account better reflects proportions of precursors observed by Northern blotting. Clearly, a decreased efficiency of CCA addition was observed in *maf1*Δ cells. Upon the shift to stress conditions the relative ratio of the form with full CCA in *maf1*Δ is much lower as compared to wild type, for all analysed tRNA precursors. Most pronounced differences in the relative amounts with full CCA were observed for tK(UUU); (9% in *maf1*Δ compared to 27% in wild type), tL(CAA) (22% to 72%) and for tW(CCA) (32% to 84%) (Fig. 2). The proportions of forms with single C or no nucleotide added at 3′ end were accordingly increased in *maf1*Δ mutant. Altogether, these data indicate less efficient CCA addition in cells lacking Maf1 under stress.

### 3.3 Effect of Maf1 on CCA addition to tRNA precursors

**Fig. 3:**
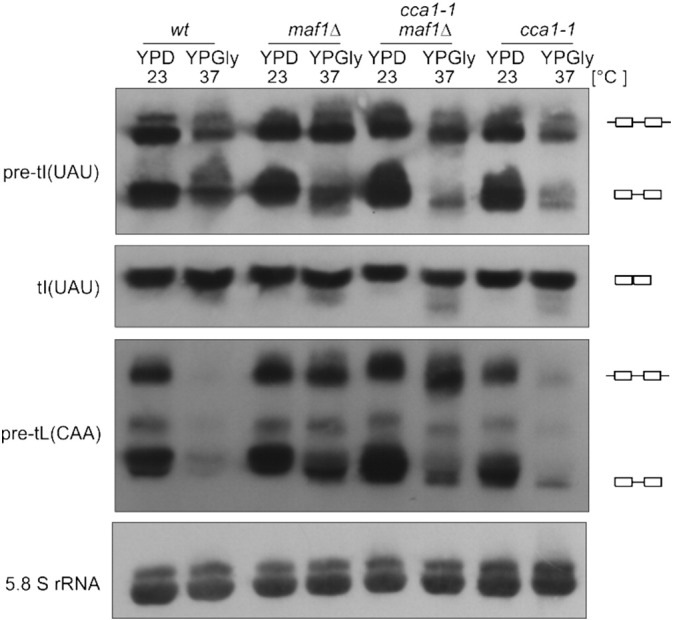
Pattern of tRNA processing in *maf1*Δ and *cca1-1* mutants. Cells were grown in YPD at 23 °C to exponential phase, than shifted for 2 h to YPGly at 37 °C, RNA isolated from harvested cells was analysed by Northern blotting with tRNA-specific probes. 5.8S rRNA was used as a loading control.

Encouraged by the results from high-throughput sequencing we further explored the effect of Maf1 on addition of CCA to 3′ termini of pre-tRNAs. First, we compared pre-tRNA profiles in *maf1*Δ and *cca1-1*, a temperature sensitive mutant of the *CCA1* gene, which encodes tRNA CCA-nucleotidyltransferase. At the non-permissive temperature CCA modification of 3′ end is decreased both for mature tRNAs and for pre-tRNAs. Northern analysis of pre-tL(CAA) identifies only single, slower-migrating band in *cca1-1* mutant shifted to restrictive conditions (37 °C YPGly), possibly representing the major precursor form of this tRNA in *maf1*Δ and *maf1*Δ *cca1* cells grown in the same conditions (Fig. 3, upper panel). Hybridization with the tI(UAU) probe provides similar observation (Fig. 3, lower panel) leading to the conclusion that the slower migrating form in *maf1*Δ mutant grown with a shift to the restrictive conditions corresponds to pre-tRNA with undermodified CCA.

**Fig. 4:**
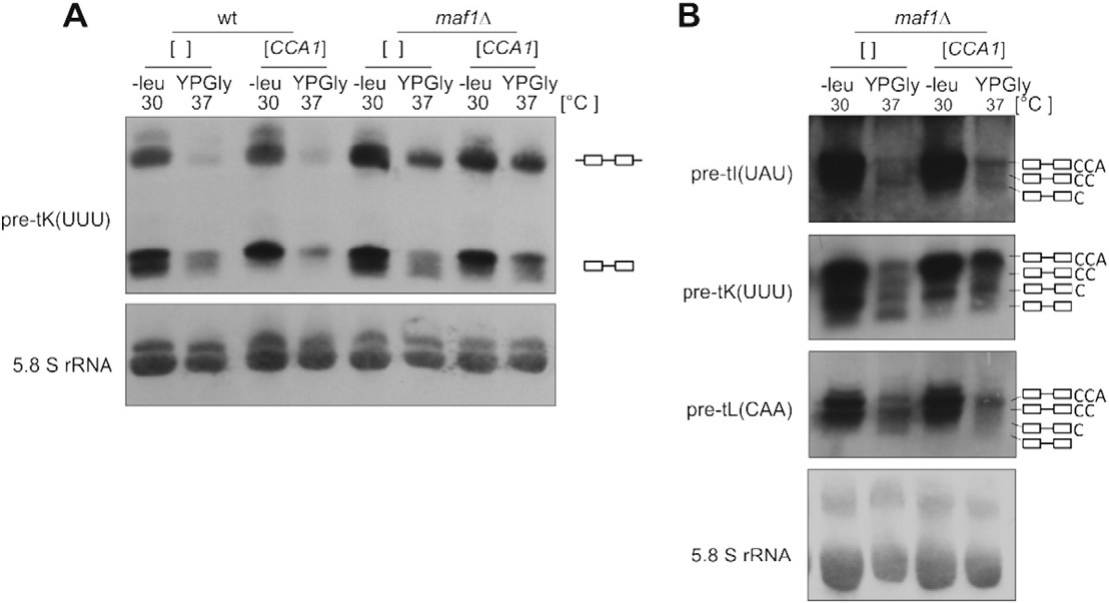
Overexpression of CCA1 gene partially restores proportion between C, CC and CCA forms altered in *maf1*Δ mutant. Wild type and *maf1*Δ cells were transformed with multicopy plasmid containing *CCA1* gene or control pRS425 vector. Transformants were grown in SC-leu to exponential phase then shifted for 2 h to YPGly at 37 °C. RNA was isolated from harvested cells and resolved on polyacrylamide gels, followed by Northern analysis with tRNA-specific probes (A). For better visualization of C, CC and CCA forms, RNA was analysed on sequencing gels followed by Northern hybridization (B). 5.8S rRNA was used as a loading control.

To support this conclusion we assessed whether the defect in tRNA processing observed in *maf1*Δ could be reversed by CCA overproduction. Cells harboring a multicopy plasmid derived from the Yeast Genomic Tiling Collection (GE), (Jones et al., 2008) with *CCA1* gene or control empty vector pRS425 were grown in minimal medium lacking leucine to exponential phase at 30 °C followed by shift to a complete glycerol containing medium for 2 h at 37 °C. RNA was isolated from harvested cultures and analysed by Northern blotting (Fig. 4A). To better distinguish between intron-containing precursors with extra added C, CC, or CCA, prior to Northern analysis RNA was resolved on sequencing gel (Fig. 4B).

Clearly, transformation of *maf1*Δ cells with *CCA1* multicopy plasmid led to an increased proportion of the form with full CCA. This confirms that regulation of tRNA transcription by Maf1 indeed plays significant role in efficiency of CCA addition to tRNA precursors.

## 4 Discussion

tRNAs are essential components of the translation machinery and therefore their biosynthesis is tightly coupled to cell growth and metabolism. A mechanism for this coordination is provided by Maf1, a general negative regulator of RNA polymerase III transcription (Pluta et al., 2001). Maf1 is essential for down-regulation of tRNA transcription during a transition from fermentative to glycerol-based medium (Ciesla et al., 2007). Both primary transcripts and end-processed intron-containing tRNA precursors were abnormally abundant in the absence of Maf1. Our previous study excluded, however, direct role of Maf1 in tRNA maturation suggesting saturation of processing machinery by the increased amounts of primary transcripts. One reason why end-matured intron-containing pre-tRNAs accumulate in *maf1 ∆* cells is the saturation of the tRNA exportin, Los1 (Karkusiewicz et al., 2011). Moreover, overproduced tRNAs are hypomodified due to the saturation of dimethyltransferase, Trm1 (Arimbasseri et al., 2015). We assumed, however, that other components of the processing pathway might be limiting when tRNA transcription is abnormally high in *maf1 ∆* upon the shift to repressive conditions.

We thus scrutinized patterns of tRNA precursors in *maf1 ∆* by using high resolution gels for Northern hybridization with probes specific for various tRNA isotypes. One band specific for *maf1 ∆* grown with a shift to the repressive conditions but not observed in control cells under the same conditions drew our attention, suggesting altered efficiency of CCA addition to end-matured intron-containing precursor of tI(UAU). This observation encouraged us to sequence precursors of tI(UAU) and four other intron-containing tRNAs on a global scale. The RNA-Seq experiment confirmed that efficiency of 3′ end modification in tRNA precursors by CCA is lower in *maf1 ∆* mutant, especially when grown in repressive conditions.

The CCA sequence is added to the tRNA 3′ termini in nuclei once the 3′ trailer sequence is released. The CCA-adding enzyme, [ATP(CTP):tRNA nucleotidyltransferase] Cca1, operates without a nucleic acid template and uses CTP and ATP as substrates. To ensure that CCA addition occurs in an error-free manner, Cca1 must switch the nucleotide specificity from C to A after the addition of two C nucleotides and stop polymerization exactly after CCA (Wilusz, 2015). Following CCA addition, the 3′ end of pre-tRNA becomes more resistant to nucleolytic cleavage (Phizicky and Hopper, 2010).

The main role of Cca1 is to generate the amino acid attachment site, so the enzyme is essential for viability. Although in bacteria, such as *E. coli*, CCA is encoded in tRNA genes, they still express the CCA-adding enzyme and its inactivation impairs cell growth. This is because the enzyme reconstructs the CCA end of partially trimmed tRNAs (Zhu and Deutscher, 1987). In yeast, the structural instability of tRNA causes addition of the double CCACCA sequence by Cca1 enzyme, which blocks aminoacylation and triggers RNA decay (Kuhn et al., 2015, Wilusz et al., 2011). By adding CCA or CCACCA, the Cca1 enzyme is thus able to elegantly control the expression and fate of tRNAs: CCA addition generates the amino acid attachment site, while CCACCA marks tRNAs for degradation. Importantly, yeast Cca1 is implicated in a tRNA nuclear export pathway which is independent on Los1 (Feng and Hopper, 2002). The Cca1 protein shuttles between the nucleus and the cytoplasm providing the potential to function as an exporter or an adapter in this tRNA nuclear export pathway. On the other hand, it has been shown previously that Los1 exportin preferentially binds tRNAs with mature 5′ termini and mature 3′ CCA-containing ends [reviewed in (Hopper and Huang, 2015, Huang and Hopper, 2016)]. Thus, if the 3'terminus is not fully modified it will be less efficiently bound to Los1 and exported by Los1 pathway. The results presented here implicate a decreased Cca1 activity in *maf1 ∆* mutants upon the shift to unfavourable growth conditions. The decreased activity of Cca1 could contribute to the accumulation of pre-tRNA in the nuclei of *maf1*Δ cells, which has been reported previously (Karkusiewicz et al., 2011). These changes in pre-tRNA cellular dynamics can have effects on programmed shifts in translation (see below).

One paradoxical phenotype of *maf1 ∆* is a decrease in efficiency of tRNA-mediated suppression, despite a global increase in tRNA levels. Non-functional suppressor tRNA reflects down regulation of translation, which might be due to general translation or due to effects on specific mRNAs whose cognate-codon bias make them differentially susceptible (Begley et al., 2007). The simplest hypothesis is that certain tRNAs transcribed in *maf1 ∆* cells are incompletely processed, hypomodified or they fail to be appropriately delivered to ribosomes. A long-term conundrum: why tRNAs overproduced in the absence of Maf1 are not fully functional has been recently solved (Arimbasseri et al., 2015) by showing that the saturation of the dimethyltransferase Trm1 plays a crucial role in the mechanism by which Maf1 affects tRNA suppression. However, reversal of antisuppression by overproduced Trm1 is incomplete, suggesting that other factors involved in tRNA maturation might also be limiting in the context of increased tRNA synthesis in *maf1*Δ (Arimbasseri et al., 2015). Results presented here implicate that Cca1 enzyme might be one of such factors.

## Figures and Tables

**Table 1:** Relative numbers of reads aligned to each pre-tRNA isotype and having extra nucleotides at their 3′ ends. Reads were subdivided to oligoA (which have at least one extra adenine at 3′ end), and reads for 3′ end processed pre-tRNA with: C, CC and CCA (added by CCA-nucleotidyltransferase) or “-” with no extra nucleotides at the 3′ termini of processed 3′ end. The number of reads and proportion in each category (in %) were given for the cDNA libraries analysed, from wild type and mutant yeast strains grown under standard conditions in glucose medium (YPD, 30 °C) or with a shift to repressive conditions (YPGly, 37 °C).

tRNA isotype	Extra nucleotides at 3′ end	Reads	wt		*maf1*Δ		*rex1*Δ	
			YPD 30 °C	YPGly 37 °C	YPD 30 °C	YPGly 37 °C	YPD 30 °C	YPGly 37 °C
tI(UAU)		Total	135,474	340,814	98,916	115,786	182,691	748,459
		% of all						
	Oligo A	(%)	14.3	13.4	13.9	22.6	19.4	16.3
	C		2.7	10.2	1.6	9.9	1.1	1.9
	CC		5.2	15.1	6.3	7.2	1.0	0.9
	CCA		12.1	9.5	19.8	3.0	6.2	4.9
	–		0.0	0.1	0.0	0.1	0.0	0.0
tF (GAA)		Total	48,710	17,533	38,622	83,422	94,244	38,118
		% of all						
	Oligo A	(%)	10.9	8.6	6.6	5.4	7.7	12.8
	C		1.2	2.4	1.4	1.2	6.4	0.9
	CC		3.3	8.5	2.9	8.6	5.0	1.8
	CCA		2.5	3.9	3.9	2.4	2.2	6.2
	–		0.9	1.3	0.8	0.7	0.5	0.4
tK(UUU)		Total	578,382	348,689	307,017	321,958	427,862	458,097
		% of all						
	Oligo A	(%)	7.9	20.8	13.7	27.7	11.0	8.9
	C		0.8	1.9	1.5	4.0	0.5	3.2
	CC		1.1	4.3	1.2	1.4	0.2	1.5
	CCA		44.0	3.3	1.8	1.7	1.2	3.5
	–		0.1	0.9	0.4	10.1	0.2	0.6
tL(CAA)		Total	2,339,638	2,450,426	517,398	1,946,043	669,469	406,151
		% of all						
	Oligo A	(%)	2.4	4.1	3.5	7.7	8.3	16.7
	C		1.9	5.6	1.4	7.6	2.0	3.3
	CC		33.9	12.0	10.6	36.9	0.6	2.4
	CCA		29.4	51.4	55.4	12.9	62.0	33.3
	–		0.4	0.4	0.3	0.6	0.7	1.0
tW(CCA)		Total	622,499	2,033,185	450,514	858,116	486,535	1,070,615
		% of all						
	Oligo A	(%)	10.2	18.1	6.1	28.6	15.0	26.7
	C		0.4	0.3	0.7	5.6	0.9	2.4
	CC		1.8	1.1	6.4	2.9	0.3	1.1
	CCA		17.0	12.6	25.7	4.5	26.0	25.1
	–		0.7	0.2	0.2	0.9	0.6	1.4

## Appendix A Supplementary data

Supplementary materials. Image 3
