# Supplementary material for: Maf1-mediated regulation of yeast RNA polymerase III is correlated with CCA addition at the 3′ end of tRNA precursors
Source: Gene. 2017 May 15;612:12–8. doi: 10.1016/j.gene.2016.08.033 (PMC5390780; doi:10.1016/j.gene.2016.08.033)
Supplement: Supplementary file 1 — Supplementary materials. [file mmc1.docx]

**SUPPLEMENTAL MATERIALS:**

**Table S1.**

| tRNA isotype | tRNA precursor type | reads | wt | | *maf1Δ* | | *rex1Δ* | |
| --- | --- | --- | --- | --- | --- | --- | --- | --- |
|  |  |  | YPD 30°C | YPGly 37°C | YPD 30°C | YPGly 37°C | YPD 30°C | YPGly 37°C |
| tI(UAU) |  | total  % of all | 135 474 | 340 814 | 98 916 | 115 786 | 182 691 | 748 459 |
|  | 3’ end processed | (%) | 21,4 | 37,9 | 29,7 | 23,4 | 10,2 | 12,8 |
|  | with 3’ trailer |  | 60,7 | 49,8 | 61,6 | 61,7 | 83,2 | 83,0 |
|  | trimmed tRNA |  | 17,9 | 12,3 | 8,7 | 14,9 | 6,6 | 4,2 |
| tF (GAA) |  | total  % of all | 48 710 | 17 533 | 38 622 | 83 422 | 94 244 | 38 118 |
|  | 3’ end processed | (%) | 9,9 | 18,3 | 10,4 | 13,4 | 16,8 | 15,7 |
|  | with 3’ trailer |  | 85,3 | 73,1 | 83,3 | 81,1 | 78,1 | 80,0 |
|  | trimmed tRNA |  | 4,7 | 8,6 | 6,4 | 5,5 | 5,2 | 4,3 |
| tK(UUU) |  | total  % of all | 578 382 | 348 689 | 307 017 | 321 958 | 427 862 | 458 097 |
|  | 3’ end processed | (%) | 52,1 | 12,1 | 5,6 | 19,1 | 2,7 | 12,2 |
|  | with 3’ trailer |  | 43,2 | 83,0 | 90,3 | 72,9 | 95,0 | 84,6 |
|  | trimmed tRNA |  | 4,7 | 4,9 | 4,1 | 8,0 | 2,3 | 3,2 |
| tL(CAA) |  | total  % of all | 2 339 638 | 2 450 426 | 517 398 | 1 946 043 | 669 469 | 406 151 |
|  | 3’ end processed | (%) | 67,1 | 71,7 | 70,4 | 59,3 | 68,8 | 49,8 |
|  | with 3’ trailer |  | 16,3 | 16,5 | 13,8 | 24,2 | 17,6 | 32,2 |
|  | trimmed tRNA |  | 16,6 | 11,8 | 15,8 | 16,5 | 13,7 | 18,1 |
| tW(CCA) |  | total  % of all | 622 499 | 2 033 185 | 450 514 | 858 116 | 486 535 | 1 070 615 |
|  | 3’ end processed | (%) | 21,4 | 15,0 | 34,3 | 14,6 | 29,0 | 32,8 |
|  | with 3’ trailer |  | 63,4 | 77,1 | 57,2 | 72,2 | 62,4 | 55,3 |
|  | trimmed tRNA |  | 15,1 | 7,8 | 8,5 | 13,3 | 8,7 | 12,0 |

**Summary of all reads from high-throughput sequencing aligned to tested pre-tRNAs.**

All reads aligned to tRNA genes were subdivided onto three classes with a respect to 3’-end:

end-processed, end unprocessed (with 3’ trailer) and uncomplete tRNA molecules (trimmed tRNA). The amount of each class was presented as a percentage of all reads mapped to given pre-tRNA

**Table S2**

**Oligonucleotides used for preparation of libraries used for Illumina sequencing**

| **Name** | **Usage** | **Sequence (5`→3`)** |
| --- | --- | --- |
| 3’**-**tRNA adapter | Ligation | 5’P**-**CUGCUGAGAUCGGAAGAGCGUCGU**-**3C |
| RT/PCR primer 1 | RT/1**-**st PCR | CTACACGACGCTCTTCCGATCT |
| tF(GAA)N | 1**-**st PCR | CTTCGGTCAAGTCATCTG |
| tI(UAU)L | 1**-**st PCR | GAAGTTTCTGTGCCAAAGAC |
| tK(UUU)D | 1**-**st PCR | GCGCATTTGCTTAAGCAAGG |
| tL(CAA)A | 1**-**st PCR | CTTGACCGCAGTGAACTGTG |
| tW(CCA)G1 | 1cst PCR | CTTGGAAATTCCACGGAATAAGATTGC |
| tF(GAA)N ext | 2**-**nd PCR | GTGACTGGAGTTCCTTGGCACCCGAGAATTCCACTTCGGTCAAGTCATCTG |
| tI(UAU)L ext | 2**-**nd PCR | GTGACTGGAGTTCCTTGGCACCCGAGAATTCCAGAAGTTTCTGTGCCAAAGAC |
| tK(UUU)D ext | 2**-**nd PCR | GTGACTGGAGTTCCTTGGCACCCGAGAATTCCAGCGCATTTGCTTAAGCAAGG |
| tL(CAA)A ext | 2**-**nd PCR | GTGACTGGAGTTCCTTGGCACCCGAGAATTCCACTTGACCGCAGTGAACTGTG |
| tW(CCA)G1 ext | 2**-**nd PCR | GTGACTGGAGTTCCTTGGCACCCGAGAATTCCACTTGGAAATTCCACGGAATAAGATTGC |
| 2**-**nd PCR primer | 2**-**nd PCR | ACACTCTTTCCCTACACGACGCTCTTCCGATCT |
| Universal 5’ final  PCR | final PCR | AATGATACGGCGACCACCGAGATCTACACTCTTTCCCTACACGACGCTCTTCCGATCT |
| RPI**-**1 | final PCR | CAAGCAGAAGACGGCATACGAGATCGTGATGTGACTGGAGTTCCTTGGCACCCGAGAATTCCA |
| RPI**-**2 | final PCR | CAAGCAGAAGACGGCATACGAGATACATCGGTGACTGGAGTTCCTTGGCACCCGAGAATTCCA |
| RPI**-**3 | final PCR | CAAGCAGAAGACGGCATACGAGATGCCTAAGTGACTGGAGTTCCTTGGCACCCGAGAATTCCA |
| RPI4 | final PCR | CAAGCAGAAGACGGCATACGAGATTGGTCAGTGACTGGAGTTCCTTGGCACCCGAGAATTCCA |
| RPI**-**5 | final PCR | CAAGCAGAAGACGGCATACGAGATCACTGTGTGACTGGAGTTCCTTGGCACCCGAGAATTCCA |
| RPI**-**6 | final PCR | CAAGCAGAAGACGGCATACGAGATATTGGCGTGACTGGAGTTCCTTGGCACCCGAGAATTCCA |
